# Novel Pathogenic Variants Leading to Sporadic Amyotrophic Lateral Sclerosis in Greek Patients

**DOI:** 10.3390/genes15030309

**Published:** 2024-02-28

**Authors:** Ouliana Ivantsik, Anne John, Kyriaki Kydonopoulou, Konstantinos Mitropoulos, Spyridon Gerou, Bassam R. Ali, George P. Patrinos

**Affiliations:** 1 Laboratory of Pharmacogenomics and Individualized Therapy, Division of Pharmacology and Biosciences, Department of Pharmacy, School of Health Sciences, University of Patras, 26504 Rion, Greece; 2 Department of Genetics and Genomics, College of Medicine and Health Sciences, United Arab Emirates University, Al-Ain P.O. Box 15551, United Arab Emirates; 3 ANALYSI Biomedical Laboratories S.A., 54623 Thessaloniki, Greece; 4 Department of Histology and Embryology, School of Medicine, National and Kapodistrian University of Athens, 10679 Athens, Greece; 5 ASPIRE Abu Dhabi Precision Medicine Ρesearch Institute, Al-Ain P.O. Box 15551, United Arab Emirates; 6 Zayed Center for Health Sciences, United Arab Emirates University, Al-Ain P.O. Box 15551, United Arab Emirates; 7 Clinical Bioinformatics Unit, Department of Pathology, Faculty of Medicine and Health Sciences, Erasmus University Medical Center, 3000 CA Rotterdam, The Netherlands

**Keywords:** amyotrophic lateral sclerosis, sporadic ALS, genetics, *SOD1*, *FUS*, *TARDBP*, genomic variants

## Abstract

Amyotrophic lateral sclerosis (ALS) is a rapidly progressive disease that affects motor neurons, leading to paralysis and death usually 3–5 years after the onset of symptoms. The investigation of both sporadic and familial ALS highlighted four main genes that contribute to the pathogenesis of the disease: *SOD1*, *FUS*, *TARDBP* and *C9orf72*. This study aims to provide a comprehensive investigation of genetic variants found in *SOD1*, *FUS* and *TARDBP* genes in Greek sporadic ALS (sALS) cases. Our sequencing analysis of the coding regions of the abovementioned genes that include the majority of the variants that lead to ALS in 32 sALS patients and 3 healthy relatives revealed 6 variants in *SOD1*, 19 variants in *FUS* and 37 variants in *TARDBP*, of which the *SOD1* p.D90A and the *FUS* c.*356G>A (rs886051940) variants have been previously associated with ALS, while two novel nonsense pathogenic variants were also identified, namely FUS p.R241* and TDP-43 p.Y214*. Our study contributes to the worldwide effort toward clarifying the genetic basis of sALS to better understand the disease’s molecular pathology.

## 1. Introduction

Amyotrophic lateral sclerosis (ALS), also known as Lou Gehrig’s syndrome, is the third most common neurodegenerative disorder after Alzheimer’s and Parkinson’s [1]. More recent studies report an incidence between 0.6 and 3.8 per 100,000 person-years [2,3,4,5,6,7,8,9], with studies in Europe reporting 2.1–3.8 per 100,000 person-years [2,3,4,8]. The main feature of the disease is the progressive paralysis of the motor neurons. Symptoms include a focal onset of weakness, which gradually progresses to affect all the limbs and bulbar muscles, and hyperreflexia [10,11]. The disease progresses rapidly, leading to paralysis and death from respiratory failure, usually 3 to 5 years after the onset of symptoms. However, to date, there is no cure, or any presymptomatic testing available [12].

Most ALS cases (about 90–95%) are sporadic (sALS) and seem to be influenced by environmental factors but also largely by genetics, while 5–10% are familial (fALS) and mostly show an autosomal dominant pattern of inheritance [13]. The two forms have common symptoms and progression processes. After studying both familial and sporadic cases, more than 20 genes have emerged as being involved in the occurrence of ALS [14]. The four most commonly mutated genes in ALS are *SOD1*, *FUS*, *TARDBP* and *C9orf72*.

The first gene associated with the disease is *SOD1*, which encodes the enzyme superoxide dismutase (SOD1). The enzyme catalyzes the conversion of harmful superoxide radicals to nitric oxide (NO) in the superoxide anion form (ONOO-) [15,16,17]. The main mechanism proposed is that pathogenic variants in this gene can disrupt this free radical elimination process, causing their accumulation in nerve cells and death [18]. Recently, the development of animal models with a disruption in SOD1 highlighted other mechanisms, including excitotoxicity, oxidative stress, mitochondrial dysfunctions and non-cell autonomous toxicity [19]. More than 170 *SOD1* variants have been reported to date, affecting approximately 20% of fALS and 1% of sALS cases [20]. Most genomic variants are missense and span the entire coding sequence.

Another gene that is strongly associated with ALS is *FUS*, also known as translocated in liposarcoma (TLS), which encodes a protein involved in RNA processing. More than 170 genomic variants have been identified, most of them missense [21], and are associated with approximately 5% of fALS [22] and 0.3% of sALS cases [23]. It has been reported that patients with *FUS* variants have an earlier onset of symptoms than other forms of the disease, usually before the age of 45 years [24,25]. Pathogenic variants in this gene have been found to correlate with all the cellular functions that are disrupted in degenerating motor neurons [26].

In 2006, a study showed that many sALS patients have an accumulation of ubiquitinated TDP-43 protein in pathological cytosolic inclusions [27]. TDP-43 is a 43 kDa protein encoded by the *TARDBP* gene, which is involved in RNA-related processes, namely mRNA metabolism, transcription, and splicing and transport, as well as microRNA biosynthesis [28]. To date, more than 40 genomic variants in the *TARDBP* gene have been documented, almost all of them missense. Pathogenic variants in the *TARDBP* gene are responsible for ~5% of fALS [27] and 1% of sALS cases [29].

An important landmark study in understanding the genetic basis of ALS is the discovery of the GGGGCC hexanucleotide repeat expansion in the C9orf72 gene, which appears to be the most common genetic cause of the disease in Europe and North America [30,31], representing 45–50% of fALS and 5–10% of sALS cases [32]. The physiological function of C9orf72 remains unknown.

The genetic basis of ALS in the Greek population has been poorly studied. The first study was published in 2012, where the frequency of the expansion of the hexanucleotide GGGGCC in the C9orf72 gene was investigated in 146 patients with ALS [33]. In 2017, another study performed a whole-genome sequencing analysis of Greek patients and highlighted the association of variants in the FTO gene with sALS [34]. Here, we attempt to provide a comprehensive molecular genetic analysis of variants in the *SOD1*, *FUS* and *TARDBP* genes in Greek sALS patients, providing further insights on the molecular genetic spectrum of ALS in Greece.

## 2. Materials and Methods

### 2.1. Study Population

sALS patients and healthy relatives, mostly referred to from the Greek ALS patients Association and the ALS Find-a-Cure Association, were recruited from June 2010 until June 2023 at the Laboratory of Pharmacogenomics and Individualized Therapy, Department of Pharmacy, University of Patras School of Health Sciences, Greece. The study was approved by the University of Patras Bioethics Committee. All participants signed an informed consent for blood sample storage, DNA extraction and analysis. In total, the study included 32 sALS patients diagnosed according to the El Escorial criteria [35] and 3 healthy relatives (Supplementary Tables S2 and S3).

### 2.2. Genetic Testing

DNA was extracted from whole blood samples using standard laboratory procedures [34]. The concentration and quality of the DNA assessed using Nanodrop 2000 (Thermo Fisher Scientific, Waltham, MA, USA) and 50 ng of genomic DNA was used, through Polymerase Chain Reaction (PCR), to amplify 17 DNA fragments [4 fragments including exons 2–5 of *SOD1* (NM_000454.5, hg_38), 7 fragments including exons 2, 3, 5, 6, 12–15 and the 3′UTR of *FUS* (NM_004960.4, hg_38) and 6 fragments including exons 1–6 of the *TARDBP* gene (NM_007375.4, hg_38)]. The selection of these segments for analysis was based on the fact that these are the exons in which more disease-associated variants have been identified [21,36]. The amplification conditions and primer sequences are available upon request. Subsequently, PCR-amplified samples were Sanger sequenced and analyzed using the BioEdit Sequence Alignment Editor [37].

### 2.3. In Silico Analysis

For the in silico prediction of the variants’ pathogenicity we used the MutationTaster2021 [38], while missense variants were also assessed via PolyPhen-2 [39] and SIFT [40]. Known variants were also searched for in the ClinVar [41] and ClinGen [42] databases.

## 3. Results

### 3.1. SOD1

We sequenced exons 2–5 of the *SOD1* gene and found six genetic variants in three patients (Table 1). Only one of them had an assigned rsID and was classified as benign/likely benign in ClinVar and ClinGen. Three of them were silent, while the others were missense. Five of the variants were cut during the post-translational modifications, and only one (c.349A>C, p.D11A) remained in the mature functional protein. MutationTaster assessed all the variants as disease causing. PolyPhen-2 tool and SIFT assessed as “probably damaging” and “affect protein function”, respectively the missense variants (c.251T>G p.C57W score = 1; c.292A>T p.H71L; score = 0.990) that are not participating in the final functional protein, while the only variant that remains was characterized as “benign” and “tolerated” (c.349A>C p.D90A) (Table 1 and Table S1).

### 3.2. FUS

The sequencing of the selected exons of the *FUS* gene revealed several genetic variations in Greek sALS patients (Table 2). Most of the variants (n = 8) were located in the 3′ UTR, following exon 6 (n = 6), exon 3 (n = 4) and exon 2 (n = 1). We found no variants in exons 5, 12, 13, 14 or 15. Regarding variants in coding sequence, eight of them were missense, one was silent and one was nonsense. RsIDs were found for 7 of 19 variants. We searched those in ClinVar and ClinGen but found reports only for three: rs80301724 and rs151073460 classified as benign/likely benign and rs886051940 as uncertain significance. The most common variant was c.223C>A, a silent variant, found in 10 patients; 5 were heterozygous and 5 homozygous. Other common variants were c.806C>T, a missense variant found in five heterozygous patients; c.*356G>A in 3′ UTR, found in five heterozygous patients; c.626A>C, a missense variant found in three heterozygous patients; c.760C>G, a silent variant found in two heterozygous patients; and c.800A>T, a nonsense variant found in two heterozygous patients.

We were able to assess all but two variants using MutationTaster (c.*370A>T, c.*406G>A). Eleven variants were characterized as disease causing, while six, as polymorphisms. The eight missense variants were assessed via PolyPhen-2 and SIFT. SIFT characterized all variants as affecting protein function. PolyPhen-2 characterized only four of them as “probably damaging” (c.162G>T p.S28I score = 0.868; c.184G>C p.Q35H score = 0.998; c.221G>T p.G48C score = 0.999; c.830G>T p.G251C score = 1; Table 2 and Table S1).

**Figure 1:**
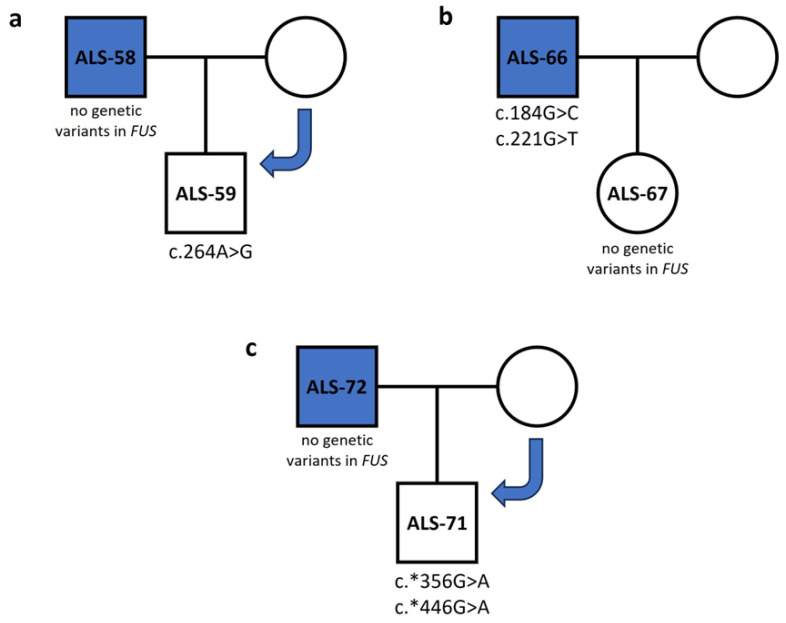
Inheritance tree diagrams of three patient–healthy relative pairs show the inheritance pattern of *FUS* variants. (**a**) Variant c.264A>G was found in healthy individual ALS-59, but not patient ALS-58. (**b**) Variants c.184G>C and c.221G>T were found in patient ALS-66 but not in their healthy daughter ALS-67. (**c**) Variants c.*356G>A and c.*446G>A were found in healthy individual ALS-71, but not in patient ALS-72.

We also sequenced the first-degree relatives of patients ALS-58, ALS-66 and ALS-72. Patient ALS-58 had no variants in the *FUS* gene, while his healthy son (ALS-59) had a missense variant (c.264A>G p.N63S) (Supplementary Tables S1 and S2). Therefore, ALS-59, a healthy individual, inherited the variant from his healthy mother, so it is probably a non-harmful variant in the *FUS* gene (Figure 1a). Patient ALS-66 had two missense variants in the *FUS* gene (c.184G>C p.Q35H; c.221G>T p.G48C), both characterized as probably damaging by in silico tools. His healthy daughter (ALS-67) had no variants in the *FUS* gene (Figure 1b, Supplementary Tables S1 and S2), a finding that suggests that c.184G>C (p.Q35H) and c.221G>T (p.G48C) may play causative roles in developing ALS. Patient ALS-72 had no variants in the *FUS* gene, but his son had two variants in the 3′ UTR of the *FUS* gene (c.*356G>A; c.*446G>A). Since his father did not have these variants, we assume that he inherited them from his healthy mother (Figure 1c).

### 3.3. TARDBP

The sequencing of *TARDBP* exons revealed 37 genetic variations (Table 3), scattered throughout the whole coding sequence. In total, 22 of the variants were missense, 10 were silent, 4 variants were found in the UTRs, and we also identified 1 nonsense variant causing the preliminary termination of translation in amino acid 214. A total of 11 variants were found in exon 6, containing the 3′ UTR; 10 variants in exon 5 (n = 10); 7 variants in exon 2; 6 variants in exon 3; 2 variants in exon 1, containing the 5′ UTR; and 1 variant in exon 4. Only five variants had assigned rsIDs (Table 3). ClinVar and ClinGen report the silent variant rs61730366 as benign/likely benign, while the missense variant rs1643653768 as likely pathogenic or uncertain significance.

The most common variants found were the missense c.274G>A p.E57K and the silent variant c.1134G>A found in eight patients. Nine of the missense variants were characterized as possibly damaging by all in silico tools: c.295G>A (p.D64N; score = 0.570), c.295G>T (p.D64Y; score = 0.904), c.490G>T (p.V129F; score = 0.494), c.500T>G (p.V132G; score = 0.943), c.741G>T (p.Q212H; score = 0.865), c.1001G>A (p.G299E; score = 0.953), c.1180C>A (p.Q326R; score = 0.654), c.1322C>T (p.S406F; score = 0.922) and c.1328C>T (p.S408F; score = 0.472). Also, PolyPhen-2 characterized five variants as “probably damaging”: c.304T>G (p.W67G; score = 0.996), c.594G>C (p.Q163H; score = 0.997), c.686T>G (p.F193L; score = 0.988), c.781A>G (p.R226G; score = 1) and c.1326G>T (p.K407N; score = 0.998) (Table 3 and Table S3).

**Figure 2:**
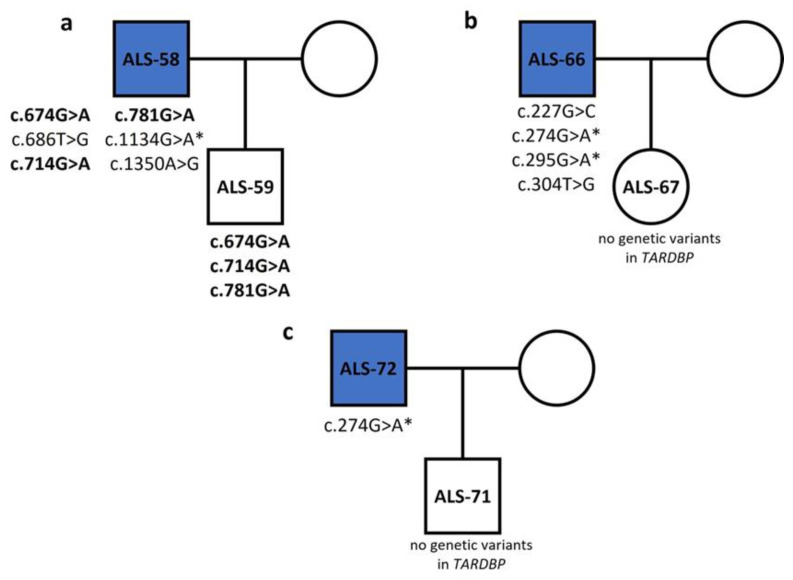
Inheritance tree diagrams of three patient–healthy relative pairs show the inheritance pattern of *TARDBP* variants. **Bold** indicates variants inherited from an ALS patient to healthy offspring. (*) indicate variants that are common in patients but not in healthy individuals.

Comparing genetic variants in patient–healthy relative pairs, we saw that healthy individual ALS-59 carried three out of six variants from his father (ALS-58): a silent variant (c.714G>A) and two missense variants (c.674G>A, c.781A>G) (Figure 2a). Based on this, we assume that the combination of these variants does not lead to ALS. Moreover, the genetic variant c.1134G>A, which was identified in the ALS-58 patient but not in his healthy son, was also identified in seven more sALS patients (Table 3). Healthy individual ALS-67 had no variants in *TARDBP*, while her father had four: c.227G>C (p.R41P), c.274G>A (p.E57K), c.295G>A (p.D64N) and c.304T>G (p.W67G) (Figure 2b). Also, ALS-71 had zero variants in *TARDBP*, but his father carried the c.274G>A variant (Figure 2c). We saw that the c.274G>A variant was found in ALS-66, ALS-72 and seven more sALS cases, but at none of the healthy offsprings, which implies that it may be a variant related to the overall sALS phenotype. Genetic variants in the 295th nucleotide were also common in patients (c.295G>A/T), but not in healthy relatives.

## 4. Discussion

The aim of this study was to investigate the genetic variations in Greek ALS patients, through molecular genetic analysis in the *SOD1*, *FUS* and *TARDBP* genes. Our sequencing analysis revealed a number of genetic variants that may contribute to ALS pathogenesis. The most significant were the missense variant c.349A>C (p.D90A) in the *SOD1* gene, a nonsense variant c.800A>T (p.R241*) in the *FUS* gene, a nonsense variant c.744C>G (p.Y214*) in the *TARDBP* gene and the c.1134G>A and c.274G>A (p.E57K) variants, which were reported only in patients and not in healthy first-degree relatives.

Regarding *SOD1*, we found six variants, half of them silent and the others missense. Five of the variants were on the part of the protein that is cut during post-translational modification, so they probably do not affect the function of the mature protein. On the other hand, all in silico analysis tools assessed the two missense variants (c.251T>G and c.292A>T) as “probably damaging”, so these may affect the protein’s folding, consequently obstructing proper post-translational modifications. Despite the fact that there have been no reported mutational hotspots in *SOD1*, we found three variants in the 248–254 region of the coding sequence (56–58 amino acids), suggesting that this may be a mutational hotspot. This region does not participate in the functional protein, but another study has reported a causative variant in amino acid 59 (p.S59S) [43]. One patient was homozygous in the c.349A>C missense variant. This was the only variant found that alters the mature protein’s sequence. This variant has been repeatedly reported before in Non-Hispanic White Americans sALS cases [44]; Caucasian, Canadian sALS and fALS cases [45], European Italian fALS cases [43]; and Asian, Iranian fALS cases [46]. PolyPhen-2 and SIFT characterized this variant as benign, but MutationTaster characterized it as disease causing. There is a family report where the c.349A>C heterozygous status was associated with ALS symptoms and related death [47]. Considering the fact that this is the sixth report highlighting the c.349A>C missense variant in ALS patients, we suggest that it is probably one of the genetic causes of both sporadic and familial ALS. Also, it would be interesting to investigate if the two missense variants that were characterized as possibly damaging affect the proper protein maturation.

In the *FUS* gene, we report 19 genetic variants. Most of them were located in 3′ UTR. Genomic variants in the 3′ UTR of the *FUS* gene have been repeatedly associated with ALS [48,49,50,51,52,53,54], but only two of the variants we found have been previously reported in ALS cases, specifically c.*41G>A (rs80301724) and c.*356G>A (rs886051940) [50,51,54,55]. The c.*41G>A (rs80301724) variant is a common variant that has been excluded as causative for ALS, since it has been proven to have the same frequency in patients and healthy controls [50,51,54]. Moreover, ClinVar and ClinGen report the variant as benign/likely benign. The rs886051940 variant found in five patients, showing a high frequency in the Greek sALS population, has been previously associated with ALS pathogenesis [55]. It is interesting that healthy individual ALS-71 also had the rs886051940 variant alongside another 3′ UTR variant, both of which he probably inherited from his healthy mother. This finding indicates that rs886051940 may be a causative variant in ALS with reduced penetrability. Based on ClinVar and ClinGen, the variant is classified as uncertain significance. The suggested mechanism on how variants in the 3′ UTR of *FUS* can cause ALS is that these variants drastically increase the FUS protein expression in the patients’ fibroblasts [54]. So, the overexpression of *FUS* seems to be toxic for nerve cells.

We also found 11 genetic variations in exons 2, 3 and 6: 8 missense, 2 silent and 1 nonsense variant. It is very interesting that most studies are referring to variants located in exons 14–15 [21], while in our study, we found none in these exons. This suggests that the genetic basis of the Greek sALS patients has a unique pattern, consistent with our previous findings. The most common variant was c.223C>A, found in 10 patients, half of them homozygous. This substitution does not alter the protein sequence, so it is interesting to investigate if it is associated with ALS or is just a common variant in the Greek population, probably as a result of a founder effect. The other silent variant (c.760C>G, rs151073460) has been previously reported in Parkinson’s disease patients; this means that despite the fact that it does not alter the protein’s sequence, it may still disrupt protein function [56]. Although ClinVar reported the variant as benign/likely benign, MutationTaster assessed it as disease causing. SIFT characterized all missense variants as probably damaging, but PolyPhen-2 characterized only four out of eight variants with this term. The other missense variants that were characterized as benign were also common in more than one sample, which contributes to the assumption that they are not harmful, but common Greek variants.

A very important finding is a novel nonsense variant (c.800A>T) that causes the early termination of translation in codon 241. This results in a truncated protein with less than half of the amino acids. The consequence of having a FUS protein that is shorter than the wild type has been studied in a mouse model with a truncated FUS mutant protein (amino acids 1–359). The mice developed ALS symptoms, but at the end stage, they showed a greater than 50% preservation of spinal motor neurons [25,57,58,59,60], suggesting that a truncated protein may be even partially sufficient for neuron function. Although, the variant we found leads to a significantly shorter protein, it may have more severe consequences in motor neurons.

*TARDBP* was the gene with the most genetic variants found in our study. Variants in this gene are common in sALS cases [29]. Although previous studies have identified variants mostly in exon 6 [61], we found variants that span the whole coding sequence. Only one of the variants we found has been recorded before in ALS cases (c.995G>T p.G297V) [62,63]. This is the first study recording variants in UTR; specifically, we found two variants in 5′ UTR (c.5T>C; c.1347G>A) and two in 3′ UTR (c.1350A>G; c.24C>G). As mentioned previously, the region most strongly associated with pathogenic variants that lead to ALS in *FUS* is 5′ UTR, as it leads to overexpression, which is toxic for nerve cells, but there is no corresponding reference for variants in *TARDBP*’s UTRs.

The two most common variants we found in *TARDBP* are c.1134G>A, which is a silent variant, and c.274G>A p.E57K, a missense variant characterized as benign via PolyPhen-2. Both of them have no or minimal influence on the protein, but they were found only in patients and not in healthy first-degree relatives. Moreover, the only variant we found that has previously been reported as associated with ALS and that both ClinVar and ClinGen classified it as both likely pathogenic and uncertain significance (c.995G>T p.G297V rs1643653768) [62,63] was also characterized as benign using the PolyPhen-2 tool; so, variants that do not significantly affect the amino acid sequence may have other consequences leading to pathogenesis, such as altering the gene’s expression. Another variant found only in patients and not in first-degree relatives is c.295G>A p.D64N, a missense variant characterized as possibly damaging by all in silico tools used.

The proposed pathogenetic mechanism by which variants in *TARDBP* lead to ALS involve the accumulation of ubiquitinated TDP-43 protein in pathological cytosolic inclusions [27]. The region of TDP-43 that interacts with UBQLN2 is amino acids 216–414 [64]. In that specific region, we found 11 genetic variants, 2 missense and probably damaging (c.781A>G p.R226G; c.1326G>T p.K407N), 5 possibly damaging [c.1001G>A (p.G299E); c.1001G>A (p.G299E); c.1180C>A (p.Q326R); c.1322C>T (p.S406F); c.1328C>T (p.S408F)] and the c.995G>T variant, which has previously been associated with ALS. This region is encoded primarily by triplets located in exon 6, where most genetic variants have been recorded [61].

Finally, we identified a nonsense variant [c.744C>G (p.Y214*)], which leads to the production of a truncated protein. Corcia and coworkers [65] also reported a truncation variant at the extreme C-terminus of the protein p.Y374*, which leads to ALS, but in our case, the preliminary termination leads to the production of a protein with half the size of the wild type.

Looking at the pattern of variants per patient (Table 1), we saw that patient ALS-30 had three variants in the *FUS* gene, one characterized via PolyPhen-2 as “probably damaging” [c.162G>T (p.S28I)] and two as “benign” [c.626A>C (p.M183L) and c.806C>T (p.R243C)]. Furthermore, patient ALS-69 had three variants in the 3′ UTR of *FUS* gene, so we assume he probably had a more severe alteration in protein expression. An even more intense accumulation of variants per patient was observed in the *TARDBP* gene. Patient ALS-57 had eight variants in the *TARDBP* gene, while ALS-58 and ALS-73 had six variants, each. The aggregation of more than one variant in the gene may have a cumulative effect, leading to a more serious disruption in protein function. Unfortunately, the clinical data of patients were not available to test this hypothesis.

### Strengths and Limitations

This study had a few strengths and limitations. This is the first study investigating the genomic variation in the three most common genes associated with sALS in the Greek population. Moreover, comparing genetic variations in patients and first-degree relatives made it possible to distinguish some of the genetic variants that are probably benign. However, the study also has several limitations. The most important is the small number of patients and healthy individuals included. There are no official data about the prevalence of ALS in Greece, but considering that it has the same prevalence as in Europe, we assumed that we included around 15–20% of the expected ALS patient population. This of course facilitated from the strong collaborative ties with the ALS patient associations throughout the years. Concerning the genetic variants we found, although there is evidence that indicates that several could lead to ALS (those characterized as probably damaging, common in patients but not in healthy relatives, etc.), we cannot draw certain conclusions. One reason is that we studied a population with common ancestry and several variants may be the results of a founder effect.

## 5. Conclusions

The genetic basis of sALS remains unclear. Even though genetic variants in the *SOD1*, *FUS* and *TARDBP* genes have been associated with ALS pathogenesis, all the variants that may lead to ALS have not been recorded, and it is not clear which variants are causative and which are benign. This study provides a thorough investigation of genetic variants found in sALS Greek patients. The study confirmed the association of several variants that were previously reported as causative, and also highlighted novel variants. The most significant novel variants are the two nonsense variants found, the c.800A>T (p.R241*) in the *FUS* gene and the c.744C>G (p.Y214*) in the *TARDBP* gene. Both variants lead to a significantly shorter protein that has ever been reported. In addition to these, we suggest that variants characterized as probably damaging and those that are common in patients but not healthy relatives should be further investigated.

ALS is a rapidly progressive disease, and there is no cure and no presymptomatic screening available. It is important that more studies investigating the genetic basis of ALS should be carried out, so we could at least gather the most common causative variants and develop a screening panel for presymptomatic assessment in high-risk individuals or help to diagnose patients faster at an early stage.

## Figures and Tables

**Table 1:** Genetic variants in the *SOD1* gene found in Greek sALS patients (variants previously associated with ALS are indicated in bold letters).

cDNA	Exon	rsID	Amino Acid Change	Functional Protein	Type of Variant	MutationTaster	PolyPhen-2	SIFT	Samples (Genotype)
c.248C>G	3	-	-	Not included	Silent	Disease causing	-	-	ALS-12 (het)
c.251T>G	3	-	p.C57W	Not included	Missense	Disease causing	Probably damaging	Affects protein function	ALS-12 (het)
c.254C>A	3	rs549580868	-	Not included	Silent	Disease causing	-	-	ALS-12 (het)
c.292A>T	3	-	p.H71L	Not included	Missense	Disease causing	Probably damaging	Affects protein function	ALS-22 (het)
c.308T>C	3	-	-	Not included	Silent	Disease causing	-	-	ALS-22 (het)
**c.349A>C**	**4**	**-**	**p.D90A**	**p.D11A**	**Missense**	**Disease causing**	**Benign**	**Tolerated**	**ALS-39 (hom)**

**Table 2:** Genetic variants in the *FUS* gene found in Greek sALS patients (variants previously associated with ALS are indicated in bold letters).

cDNA	Exon	Protein	Type of Variant	rsID	MutationTaster	PolyPhen-2	SIFT	Samples (Genotype)
c.101C>A	2	p.Q8K	Missense	-	Disease causing	Benign	Affects protein function	ALS-58 (het)
c.162G>T	3	p.S28I	Missense	-	Disease causing	Probably damaging	Affects protein function	ALS-30 (het)
c.184G>C	3	p.Q35H	Missense	rs772271532	Disease causing	Probably damaging	Affects protein function	ALS-66 (het)
c.221G>T	3	p.G48C	Missense	-	Disease causing	Probably damaging	Affects protein function	ALS-66 (het)
c.223C>A	3	-	Silent	-	Polymorphism	-	-	ALS-9 (het), ALS-12 (hom), ALS-13 (hom), ALS-14 (hom), ALS-21 (hom), ALS-24 (het), ALS-30 (het), ALS-38 (hom), ALS-40 (het), ALS-68 (het)
c.626A>C	6	p.M183L	Missense	rs762914131	Disease causing	Benign	Affects protein function	ALS-30 (het), ALS-38 (het), ALS-57 (het)
c.759G>T	6	p.G227V	Missense	-	Disease causing	Benign	Affects protein function	ALS-70 (het)
c.760C>G	6	-	Silent	rs151073460	Disease causing	-	-	ALS-10 (het), ALS-30 (het)
c.800A>T	6	p.R241*	Nonsense	-	Disease causing	-	-	ALS-38 (het), ALS-40 (het)
c.806C>T	6	p.R243C	Missense	rs1165095258	Disease causing	Benign	Affects protein function	ALS-2 (het), ALS-10 (het), ALS-22 (het), ALS-25 (het), ALS-30 (het)
c.830G>T	6	p.G251C	Missense	-	Disease causing	Probably damaging	Affects protein function	ALS-65 (het)
c.*41G>A	3′ UTR	-	-	rs80301724	Disease causing	-	-	ALS-13 (het)
c.*81C>T	3′ UTR	-	-	rs768544815	Polymorphism	-	-	ALS-73 (het)
c.*306T>C	3′ UTR	-	-	-	Polymorphism	-	-	ALS-63 (het)
c.*354A>T	**3′ UTR**	**-**	**-**	**-**	**Polymorphism**	**-**	**-**	**ALS-70 (het)**
c.*356G>A	3′ UTR	-	-	rs886051940	Polymorphism	-	-	ALS-60 (het), ALS-61 (het), ALS-64 (het), ALS-65 (het), ALS-69 (het)
c.*362T>G	3′ UTR	-	-	-	Polymorphism	-	-	ALS-73 (het)
c.*370A>T	3′ UTR	-	-	-	-	-	-	ALS-69 (het)
c.*406G>A	3′ UTR	-	-	-	-	-	-	ALS-69 (het)

**Table 3:** Genetic variants in the *TARDBP* gene found in Greek sALS patients (variants previously associated with ALS are indicated in bold letters).

cDNA	Exon	Protein	Type of Variant	rsID	MutationTaster	PolyPhen-2	SIFT	Samples (Genotype)
c.5T>C	1 (5′ UTR)	-	-	-	Disease causing	-	-	ALS-6 (het)
c.24C>G	1 (5′ UTR)	-	-	rs965172966	Polymorphism	-	-	ALS-2 (het), ALS-6 (het), ALS-13 (hom)
c.227G>C	2	p.R41P	Missense	-	Disease causing	Benign	Affects protein function	ALS-66 (het)
c.274G>A	2	p.E57K	Missense	-	Disease causing	Benign	Affects protein function	ALS-30 (het), ALS-40 (het), ALS-41 (het), ALS-66 (het), ALS-68 (het), ALS-69 (het), ALS-72 (het), ALS-73 (het)
c.295G>A	2	p.D64N	Missense	-	Disease causing	Possibly damaging	Affects protein function	ALS-41 (het), ALS-66 (het)
c.295G>T	2	p.D64Y	Missense	-	Disease causing	Possibly damaging	Affects protein function	ALS-57 (het)
c.300T>C	2	-	Silent	rs61730366	Disease causing	-	-	ALS-63 (het)
c.303C>G	2	-	Silent	-	Disease causing	-	-	ALS-57 (het)
c.304T>G	2	p.W67G	Missense	-	Disease causing	Probably damaging	Affects protein function	ALS-66 (het)
c.363G>A	3	-	Silent	-	Disease causing	-	-	ALS-73 (het)
c.405G>A	3	-	Silent	-	Disease causing	-	-	ALS-73 (het)
c.468G>T	3	p.E121D	Missense	-	Polymorphism	Benign	Tolerated	ALS-73 (het)
c.487G>A	3	p.E128K	Missense	-	Disease causing	Possibly damaging	Tolerated	ALS-73 (het)
c.490G>T	3	p.V129F	Missense	-	Disease causing	Possibly damaging	Affects protein function	ALS-73 (het)
c.500T>G	3	p.V132G	Missense	rs766116483	Disease causing	Possibly damaging	Affects protein function	ALS-25 (het)
c.594G>C	4	p.Q163H	Missense	-	Disease causing	Probably damaging	Affects protein function	ALS-41 (het)
c.674G>A	5	p.R190K	Missense	-	Disease causing	Benign	Tolerated	ALS-14 (het), ALS-58 (het)
c.686T>G	5	p.F193L	Missense	-	Disease causing	Probably damaging	Affects protein function	ALS-58 (het)
c.703G>A	5	p.D200N	Missense	-	Disease causing	Possibly damaging	Tolerated	ALS-57 (het)
c.714G>A	5	-	Silent	rs1333943256	Disease causing	-	-	ALS-58 (het)
c.715G>A	5	p.D204N	Missense	-	Disease causing	Benign	Tolerated	ALS-57 (het)
c.741G>T	5	p.Q212H	Missense	-	Disease causing	Possibly damaging	Affects protein function	ALS-14 (het)
c.744C>G	5	p.Y214*	Nonsense	-	Disease causing	-	-	ALS-57 (het)
c.777A>T	5	-	Silent	-	Disease causing	-	-	ALS-57 (het)
c.781A>G	5	p.R226G	Missense	-	Disease causing	Probably damaging	Affects protein function	ALS-57 (het), ALS-58 (het)
c.801A>T	5	-	Silent	-	Disease causing	-	-	ALS-57 (het)
c.972T>A	6	-	Silent	-	Disease causing	-	-	ALS-70 (het)
**c.995G>T**	**6**	**p.G297V**	**Missense**	**rs1643653768**	**Disease causing**	**Benign**	**Affects protein function**	**ALS-63 (het)**
c.1001G>A	6	p.G299E	Missense	-	Disease causing	Possibly damaging	Affects protein function	ALS-70 (het)
c.1134G>A	6	-	Silent	-	Disease causing	-	-	ALS-9 (het), ALS-13 (het), ALS-23 (het), ALS-25 (het), ALS-38 (het), ALS-58 (het), ALS-68 (het), ALS-73 (het)
c.1180C>A	6	p.Q326R	Missense	-	Disease causing	Possibly damaging	Affects protein function	ALS-70 (het)
c.1182G>A	6	-	Silent	-	Disease causing	-	-	ALS-70 (het)
c.1322C>T	6	p.S406F	Missense	-	Disease causing	Possibly damaging	Affects protein function	ALS-21 (het)
c.1326G>T	6	p.K407N	Missense	-	Disease causing	Probably damaging	Affects protein function	ALS-70 (het)
c.1328C>T	6	p.S408F	Missense	-	Disease causing	Possibly damaging	Affects protein function	ALS-21 (het)
c.1347G>A	6 (3′ UTR)	-	-		Disease causing	-	-	ALS-68 (het)
c.1350A>G	6 (3′ UTR)	-	-		Polymorphism	-	-	ALS-58 (het)

## Data Availability

Data are available upon request.

## Supplementary Materials

The following supporting information can be downloaded at https://www.mdpi.com/article/10.3390/genes15030309/s1: Table S1: Prediction and scores of variant pathogenicity based on computational prediction tools; Table S2: Genetic variants found in each sporadic ALS patient; Table S3: Genetic variants found in family members that do not suffer from sporadic ALS.
