# Supplementary material for: Novel Pathogenic Variants Leading to Sporadic Amyotrophic Lateral Sclerosis in Greek Patients
Source: Genes (Basel). 2024 Feb 28;15(3):309. doi: 10.3390/genes15030309 (PMC10970271; doi:10.3390/genes15030309)
Supplement: Supplementary file 1 [file genes-15-00309-s001.zip › genes-2887463-supplementary.pdf]

Supplementary Information

Novel pathogenic variants leading to sporadic amyotrophic lateral sclerosis in Greek patients

Ouliana Ivantsik [1], Anne John [2], Kyriaki Kydonopoulou [3], Konstantinos Mitropoulos [4], Spyridon Gerou [3], Bassam R. Ali [2,5] and George P. Patrinos [1,2,5,6,7,*]

[1] Laboratory of Pharmacogenomics and Individualized Therapy, Division of Pharmacology and Biosciences, Department of Pharmacy, School of Health Sciences, University of Patras, 26504 Rion, Greece.
[2] Department of Genetics and Genomics, College of Medicine and Health Sciences, United Arab Emirates University, Al-Ain, P.O. Box 15551, United Arab Emirates
[3] ANALYSI Biomedical Laboratories S.A., 54623 Thessaloniki, Greece
[4] Department of Histology and Embryology, School of Medicine, National and Kapodistrian University of Athens, 10679 Athens, Greece
[5] ASPIRE Abu Dhabi Precision Medicine Research Institute, Al-Ain, P.O. Box 15551, United Arab Emirates
[6] Zayed Center for Health Sciences, United Arab Emirates University, Al-Ain, P.O. Box 15551, United Arab Emirates
[7] Clinical Bioinformatics Unit, Department of Pathology, Faculty of Medicine and Health Sciences, Erasmus University Medical Center, 3000 CA Rotterdam, the Netherlands

Table S1. Prediction and scores of variants pathogenicity based on the MutationTaster, PolyPhen-2 and SIFT computational prediction tools.

Gene	cDNA	Protein	MutationTaster		PolyPhen-2		SIFT	
			**Prediction**	**Score**	**Prediction**	**Score**	**Prediction**	**Score**
SOD1	c.248C>G	-	Disease causing	1	-	-	-	-
SOD1	c.251T>G	p.C57W	Disease causing	1	Probably damaging	1	Affects protein function	0
SOD1	c.254C>A	-	Disease causing	1	-	-	-	-
SOD1	c.292A>T	p.H71L	Disease causing	1	Probably damaging	0.99	Affects protein function	0
SOD1	c.308T>C	-	Disease causing	1	-	-	-	-
SOD1	c.349A>C	p.D90A	Disease causing	2.09	Benign	0	Tolerated	0.07
FUS	c.101C>A	p.Q8K	Disease causing	0.62	Benign	0.01	Affects protein function	0
FUS	c.162G>T	p.S28I	Disease causing	0.97	Probably damaging	0.87	Affects protein function	0
FUS	c.184G>C	p.Q35H	Disease causing	1	Probably damaging	1	Affects protein function	0
FUS	c.221G>T	p.G48C	Disease causing	0.99	Probably damaging	1	Affects protein function	0
FUS	c.223C>A	-	Polymorphism	5.68	-	-	-	-
FUS	c.626A>C	p.M183L	Disease causing	0.74	Benign	0	Affects protein function	0
FUS	c.759G>T	p.G227V	Disease causing	1	Benign	0..39	Affects protein function	0
FUS	c.760C>G	-	Disease causing	1	-	-	-	-
FUS	c.800A>T	p.R241*	Disease causing	1	-	-	-	-
FUS	c.806C>T	p.R243C	Disease causing	1	Benign	0.02	Affects protein function	0
FUS	c.830G>T	p.G251C	Disease causing	1	Probably damaging	1	Affects protein function	0

FUS	c.*41G>A	-	Disease causing	1	-	-	-	-
FUS	c.*81C>T	-	Polymorphism	1	-	-	-	-
FUS	c.*306T>C	-	Polymorphism	1	-	-	-	-
FUS	c.*354A>T	-	Polymorphism	1	-	-	-	-
FUS	c.*356G>A	-	Polymorphism	1	-	-	-	-
FUS	c.*362T>G	-	Polymorphism	1	-	-	-	-
FUS	c.*370A>T	-	-	-	-	-	-	-
FUS	c.*406G>A	-	-	-	-	-	-	-
TARDBP	c.5T>C	-	Disease causing	1	-	-	-	-
TARDBP	c.24C>G	-	Polymorphism	1	-	-	-	-
TARDBP	c.227G>C	p.R41P	Disease causing	1	Benign	0.01	Affects protein function	0
TARDBP	c.274G>A	p.E57K	Disease causing	1	Benign	0.02	Affects protein function	0
TARDBP	c.295G>A	p.D64N	Disease causing	1	Possibly damaging	0.57	Affects protein function	0
TARDBP	c.295G>T	p.D64Y	Disease causing	1	Possibly damaging	0.9	Affects protein function	0
TARDBP	c.300T>C	-	Disease causing	1	-	-	-	-
TARDBP	c.303C>G	-	Disease causing	1	-	-	-	-
TARDBP	c.304T>G	p.W67G	Disease causing	1	Probably damaging	1	Affects protein function	0
TARDBP	c.363G>A	-	Disease causing	1	-	-	-	-
TARDBP	c.405G>A	-	Disease causing	1	-	-	-	-
TARDBP	c.468G>T	p.E121D	Polymorphism	0.81	Benign	0.01	Tolerated	0.88

TARDBP	c.487G>A	p.E128K	Disease causing	1	Possibly damaging	0.85	Tolerated	0.27
TARDBP	c.490G>T	p.V129F	Disease causing	1	Possibly damaging	0.49	Affects protein function	0.02
TARDBP	c.500T>G	p.V132G	Disease causing	1	Possibly damaging	0.94	Affects protein function	0
TARDBP	c.594G>C	p.Q163H	Disease causing	1	Probably damaging	1	Affects protein function	0.02
TARDBP	c.674G>A	p.R190K	Disease causing	1	Benign	0.11	Tolerated	0.29
TARDBP	c.686T>G	p.F193L	Disease causing	1	Probably damaging	0.99	Affects protein function	0.03
TARDBP	c.703G>A	p.D200N	Disease causing	1	Possibly damaging	0.77	Tolerated	0.21
TARDBP	c.714G>A	-	Disease causing	1	-	-	-	-
TARDBP	c.715G>A	p.D204N	Disease causing	1	Benign	0.17	Tolerated	0.08
TARDBP	c.741G>T	p.Q212H	Disease causing	1	Possibly damaging	0.87	Affects protein function	0.01
TARDBP	c.744C>G	p.Y214*	Disease causing	1	-	-	-	-
TARDBP	c.777A>T	-	Disease causing	1	-	-	-	-
TARDBP	c.781A>G	p.R226G	Disease causing	1	Probably damaging	1	Affects protein function	0
TARDBP	c.801A>T	-	Disease causing	1	-	-	-	-
TARDBP	c.972T>A	-	Disease causing	1	-	-	-	-
TARDBP	c.995G>T	p.G297V	Disease causing	1	Benign	0.05	Affects protein function	0.02
TARDBP	c.1001G>A	p.G299E	Disease causing	1	Possibly damaging	0.95	Affects protein function	0.03
TARDBP	c.1134G>A	-	Disease causing	1	-	-	-	-
TARDBP	c.1180C>A	p.Q326R	Disease causing	1	Possibly damaging	0.65	Affects protein function	0.01
TARDBP	c.1182G>A	-	Disease causing	1	-	-	-	-

TARDBP	c.1322C>T	p.S406F	Disease causing	1	Possibly damaging	0.92	Affects protein function	0
TARDBP	c.1326G>T	p.K407N	Disease causing	1	Probably damaging	1	Affects protein function	0
TARDBP	c.1328C>T	p.S408F	Disease causing	1	Possibly damaging	0.47	Affects protein function	0
TARDBP	c.1347G>A	-	Disease causing	1	-	-	-	-
TARDBP	c.1350A>G	-	Polymorphism	0.99	-	-	-	-

Table S2: Genetic variants found in each sporadic ALS patient.

Sample	Gene	Exon	Variant			Genotype	Type of variant
			cDNA	Protein	rsID		
ALS-1	No variants found						
ALS-2	*FUS*	6	c.806C>T	p.R243C	rs1165095258	het	Missense
	TARDBP	5′ UTR	c.24C>G	-	rs965172966	het	-
ALS-6	*TARDBP*	5′ UTR	c.5T>C	-	-	het	-
	TARDBP	5′ UTR	c.24C>G	-	rs965172966	het	-
ALS-9	*FUS*	3	c.223C>A	-	-	het	Silent
	TARDBP	6	c.1134G>A	-	-	het	Silent
ALS-10	*FUS*	6	c.760C>G	-	rs151073460	het	Silent
	FUS	6	c.806C>T	p.R243C	rs1165095258	het	Missense
ALS-12	*SOD1*	3	c.248C>G	-	-	het	Silent
	SOD1	3	c.251T>G	p.C57W	-	het	Missense
	SOD1	3	c.254C>A	-	rs549580868	het	Silent
	FUS	3	c.223C>A	-	-	hom	Silent
ALS-13	*FUS*	3	c.223C>A	-	-	hom	Silent
	FUS	3' UTR	c.*41G>A	-	rs80301724	het	-
	TARDBP	5′ UTR	c.24C>G	-	rs965172966	hom	-
	TARDBP	6	c.1134G>A	-	-	het	Silent

ALS-14	*FUS*	3	c.223C>A	-	-	hom	Silent
	TARDBP	5	c.674G>A	p.R190K	-	het	Missense
	TARDBP	5	c.741G>T	p.Q212H	-	het	Missense
ALS-21	*FUS*	3	c.223C>A	-	-	hom	Silent
	TARDBP	6	c.1322C>T	p.S406F	-	het	Missense
ALS-22	*SOD1*	3	c.292A>T	p.H71L	-	het	Missense
	SOD1	3	c.308T>C	-	-	het	Silent
	FUS	6	c.806C>T	p.R243C	rs1165095258	het	Missense
ALS-23	*TARDBP*	6	c.1134G>A	-	-	het	Silent
ALS-24	*FUS*	3	c.223C>A	-	-	het	Silent
ALS-25	*FUS*	6	c.806C>T	p.R243C	rs1165095258	het	Missense
	TARDBP	3	c.500T>G	p.V132G	rs766116483	het	Missense
	TARDBP	6	c.1134G>A	-	-	het	Silent
ALS-30	*SOD1*	3	c.162G>T	p.S28I	-	het	Missense
	FUS	3	c.223C>A	-	-	het	Silent
	FUS	6	c.626A>C	p.M183L	rs762914131	het	Missense
	FUS	6	c.760C>G	-	rs151073460	het	Silent
	FUS	6	c.806C>T	p.R243C	rs1165095258	het	Missense
	TARDBP	2	c.274G>A	p.E57K	-	het	Missense
ALS-38	*FUS*	3	c.223C>A	-	-	hom	Silent

	FUS	6	c.626A>C	p.M183L	rs762914131	het	Missense
	FUS	6	c.800A>T	p.R241*	-	het	Nonsense
	TARDBP	6	c.1134G>A	-	-	het	Silent
ALS-39	*SOD1*	4	c.349A>C	p.D90A	-	hom	Missense
ALS-40	*FUS*	3	c.223C>A	-	-	het	Silent
	FUS	6	c.800A>T	p.R241*	-	het	Nonsense
	TARDBP	2	c.274G>A	p.E57K	-	het	Missense
ALS-41	*TARDBP*	2	c.274G>A	p.E57K	-	het	Missense
	TARDBP	2	c.295G>A	p.D64N	-	het	Missense
	TARDBP	4	c.594G>C	p.Q163H	-	het	Missense
ALS-57	*FUS*	6	c.626A>C	p.M183L	rs762914131	het	Missense
	TARDBP	2	c.295G>T	p.D64Y	-	het	Missense
	TARDBP	2	c.303C>G	-	-	het	Silent
	TARDBP	5	c.703G>A	p.D200N	-	het	Missense
	TARDBP	5	c.715G>A	p.D204N	-	het	Missense
	TARDBP	5	c.744C>G	p.Y214*	-	het	Nonsense
	TARDBP	5	c.777A>T	-	-	het	Silent
	TARDBP	5	c.781G>A	p.R226G	-	het	Missense
	TARDBP	5	c.801A>T	-	-	het	Silent
ALS-58	*SOD1*	2	c.101C>A	p.Q8K	-	het	Missense

	TARDBP	5	c.674G>A	p.R190K	-	het	Missense
	TARDBP	5	c.686T>G	p.F193L	-	het	Missense
	TARDBP	5	c.714G>A	-	rs1333943256	het	Silent
	TARDBP	5	c.781G>A	p.R226G	-	het	Missense
	TARDBP	6	c.1134G>A	-	-	het	Silent
	TARDBP	3′ UTR	c.1350A>G	-	-	het	-
ALS-60	*FUS*	3′ UTR	c.*356G>A	-	rs886051940	het	-
ALS-61	*FUS*	3′ UTR	c.*356G>A	-	rs886051940	het	-
ALS-62	No variants found						
ALS-63	*FUS*	3′ UTR	c.*306T>C	-	-	het	-
	TARDBP	2	c.300T>C	-	rs61730366	het	Silent
	TARDBP	6	c.995G>T	p.G297V	rs1643653768	het	Missense
ALS-64	*FUS*	3′ UTR	c.*356G>A	-	rs886051940	het	-
ALS-65	*FUS*	6	c.830G>T	p.G251C	-	het	Missense
	FUS	3′ UTR	c.*356G>A	-	rs886051940	het	-
ALS-66	*FUS*	3	c.184G>C	p.Q35H	rs772271532	het	Missense
	FUS	3	c.221G>T	p.G48C	-	het	Missense
	TARDBP	2	c.227G>C	p.R41P	-	het	Missense
	TARDBP	2	c.274G>A	p.E57K	-	het	Missense
	TARDBP	2	c.295G>A	p.D64N	-	het	Missense

	TARDBP	2	c.304T>G	p.W67G	-	het	Missense
ALS-68	*FUS*	3	c.223C>A	-	-	het	Silent
	TARDBP	2	c.274G>A	p.E57K	-	het	Missense
	TARDBP	6	c.1134G>A	-	-	het	Silent
	TARDBP	3′ UTR	c.1347G>A	-	-	het	-
ALS-69	*FUS*	3′ UTR	c.*356G>A	-	rs886051940	het	-
	FUS	3′ UTR	c.*370A>T	-	-	het	-
	FUS	3′ UTR	c.*406G>A	-	-	het	-
	TARDBP	2	c.274G>A	p.E57K	-	het	Missense
ALS-70	*FUS*	6	c.759G>T	p.G227V	-	het	Missense
	FUS	3′ UTR	c.*354A>T	-	-	het	-
	TARDBP	6	c.972T>A	-	-	het	Silent
	TARDBP	6	c.1001G>A	p.G299E	-	het	Missense
	TARDBP	6	c.1180C>A	p.Q326R	-	het	Missense
	TARDBP	6	c.1182G>A	-	-	het	Silent
	TARDBP	6	c.1328C>T	p.K407N	-	het	Missense
ALS-72	*TARDBP*	2	c.274G>A	p.E57K	-	het	Missense
ALS-73	*FUS*	3′ UTR	c.*81C>T	-	rs768544815	het	-
	FUS	3′ UTR	c.*362T>G	-	-	het	-
	TARDBP	2	c.274G>A	p.E57K	-	het	Missense

	TARDBP	3	c.363G>A	-	-	het	Silent
	TARDBP	3	c.405G>A	-	-	het	Silent
	TARDBP	3	c.468G>T	p.E121D	-	het	Missense
	TARDBP	3	c.487G>A	p.E128K	-	het	Missense
	TARDBP	3	c.490G>T	p.V129F	-	het	Missense

Table S3. Genetic variants found in family members that do not suffer from sporadic ALS.

Sample	Gene	Exon	Variant			Genotype	Type of variant
			cDNA	protein	rsID		
ALS-59 (son of ALS-58)	*FUS*	3	c.264A>G	p.N63S	-	het	Missense
	TARDBP	5	c.674G>A	p.R190K	-	het	Missense
	TARDBP	5	c.714G>A	-	rs1333943256	het	Silent
	TARDBP	5	c.781G>A	p.R226G	-	het	Missense
ALS-67 (daughter of ALS-66)	No variants found						
ALS-71 (son of ALS-72)	*FUS*	3′ UTR	c.*356G>A	-	rs886051940	het	-
	FUS	3′ UTR	c.*446G>A	-	-	het	-
